# Perceptions of cannabis warnings and recommendations for improvement: a qualitative study with people who use cannabis from the United States

**DOI:** 10.1186/s12889-025-23518-1

**Published:** 2025-07-03

**Authors:** Leah M. Ranney, Sonia A. Clark, Caroline J. Meek, Kristen L. Jarman, Sarah D. Kowitt

**Affiliations:** 1 Department of Family Medicine, University of North Carolina at Chapel Hill, 590 Manning Dr. CB 7595, Chapel Hill, NC 27599 USA; 2 Center for Health Promotion and Disease Prevention, University of North Carolina at Chapel Hill, Chapel Hill, NC 27599 USA; 3 Lineberger Comprehensive Cancer Center, University of North Carolina at Chapel Hill, Chapel Hill, NC 27599 USA; 4 Department of Health Behavior, University of North Carolina at Chapel Hill, Chapel Hill, NC 27599 USA; 5 RTI International, Research Triangle Park, NC 27709 USA

**Keywords:** Cannabis warnings, Adults, Qualitative research, Health warning labels

## Abstract

**Background:**

Most states with legalized cannabis require warnings, but current cannabis warnings are unnoticed, difficult to read, and confusing. There is limited cannabis-specific research on which warning characteristics (e.g., size, content) effectively communicate the risks of cannabis use. This research explores perceptions of cannabis warning labels from a qualitative perspective.

**Methods:**

We conducted six virtual focus groups (*n* = 36 participants) in March and April 2023 with U.S. adults (21+) who reported using cannabis in the past 30 days and resided in a state with legalized recreational cannabis. After exploring risk perceptions of cannabis products, participants were shown existing warnings on cannabis packages from states that have legalized cannabis and novel warnings on cannabis packages created by the study team and asked about cognitive and affective reactions. The resulting audio recordings were transcribed, and ATLAS.ti was used to perform thematic data analysis.

**Results:**

Half of the participants identified as White, 22% as Black or African American, and 28% as another race. Participants’ mean age was 30.5 years, and, on average, participants reported using cannabis 3.7 of the past 7 days. Themes that emerged from the data were that participants perceived the purpose of warnings as accurately educating or promoting safe use: “*I would say to inform people of the health risk so they can make an educated decision.”* Participants did not notice existing warnings and wanted warnings to capture attention: *“I’ve never noticed one […]”* and *“I would want some kind of a warning icon or symbol […].”* Finally, participants were aware of both the benefits and risks of using cannabis: “*I smoke for my anxiety*” and “[…] *I’ll get some sort of like paranoia.”*

**Conclusions:**

Our findings highlight areas for cannabis warning improvement, particularly with the warning content, design, and placement. Cannabis warnings that inform, educate, and guide less risky use may be well-received by people who use cannabis. More research is needed on the health effects of cannabis use to aid in the development of evidence-based cannabis warnings, particularly around mental health concerns.

**Supplementary Information:**

The online version contains supplementary material available at 10.1186/s12889-025-23518-1.

## Introduction

In 2021, 34.9 million adults in the United States (US) used cannabis in the past month [1]. As cannabis grows in popularity, public perception of risk is declining, and many people underestimate or are unaware of the potential harms of cannabis [2–5]. This is partly due to widespread cannabis advertising and exposure [6], especially in states with legalized cannabis [7]. As a result, potential consumers often receive messages about the benefits but not the consequences of cannabis use, such as cannabis use disorder, damage to brain development (particularly for those under age 25), impaired driving, and mental health concerns, including temporary psychosis and schizophrenia [8, 9]. This imbalance is concerning since people need information on both benefits and harms to make informed decisions about whether they will use cannabis and how to do so safely.

One way to inform people about cannabis-related harms is through warning labels on packages. Warning labels are a cost-effective, population-level intervention that can increase knowledge and risk perceptions of product harms, but only if they are well-designed [10–15]. While all states with legalized cannabis require warnings, they are often placed on the back of packages, use small, hard-to-read (e.g., 6-point font) font, and are lengthy (e.g., more than 100 words) and, as a result, often go unnoticed [16]. Indeed, current evidence suggests that existing cannabis warnings in the US are ineffective, and research is needed to improve them [14, 17]. Moreover, cannabis warnings are inconsistent across US states, which may add to confusion about actual health risks [18]. Lessons learned from tobacco and alcohol warning research suggest that effective warnings should be large, ideally covering 50% of the front display, and incorporate text and images about specific, adverse health effects [19–22]. Attention-grabbing health warnings that increase cognitive and affective reactions can influence intentions and change behaviors [23, 24]. While best practices in tobacco and alcohol warning research provide a useful starting place, the unique balance of benefits and risks associated with cannabis use requires a nuanced approach to warning label development.

Two important ways to improve cannabis warnings are through warning design and warning content. In general, more visual attention is allocated to cannabis product branding and flavors than to warning labels [25, 26]. This makes the design of warning labels all the more important because they must detract attention from other package features to be acknowledged. A 2023 systematic review of cannabis warnings found that mandated warnings from the US were less effective than those from Canada on a variety of different outcomes [14]. This is likely because warnings from Canada adhere to best practices for warning label design (e.g., warnings are displayed on plain packages, warnings use contrasting color to attract attention, warnings are on the front of packages), while those from the US do not. Indeed, a 2025 online experiment showed that plain packaging can improve warning recall [27]. Another potential way to improve warning design is to implement pictorial warnings. However, at present, the evidence is mixed. One study found no differences in intentions between text-only and pictorial cannabis warnings [28], while two other studies found some beneficial effects of pictorial cannabis, with one study finding that warnings reduced appeal for adolescents [29] and another showing that pictorial cannabis warnings were more effective than other warning formats at decreasing appeal and interest, especially when presented with a yellow background [30]. 

In terms of warning content, research shows that some warning topics are less believable than others. For instance, Massey’s 2023 systematic review found that warnings on addiction were generally the least effective, while other warning topics like risks for pregnancy and brain health were perceived as more effective. However, more research is needed, especially since experiments and qualitative studies to date have not examined novel warning topics, such as chronic bronchitis or accidental ingestion by children. A study from Australia asked people who used cannabis to suggest actual warnings that could go on product packages, finding that people described a variety of different risks, including harms to mental health, operating machinery, addiction, short-term physical side effects, and the importance of responsible use [31]. However, this study did not explore how warnings should be presented (e.g., format, length) or the actual effectiveness of different warning themes [31]. 

Overall, while qualitative studies have explored risk perceptions of cannabis use, often among priority populations like youth [32] or pregnant women [33], there is limited cannabis-specific research on how to improve warning characteristics (e.g., design, content) to effectively communicate the risk of cannabis use. To contribute and expand upon previous cannabis warning research, the goal of our study was to examine how people who use cannabis perceive existing warnings, describe product risks, and react to novel cannabis warnings.

## Methods

### Participants

Participants were a sample of adults (ages 21+) who spoke English, reported using cannabis in the past 30 days, and resided in a US state that has legalized cannabis for recreational use (i.e., adult non-medical use). To recruit participants, we posted ads on Facebook and Instagram that directed potential participants to fill out an online screener to determine if they were eligible. Two members of the research team contacted eligible participants and invited them to participate in an online 15-minute video call using Zoom software to confirm eligibility criteria, obtain consent, practice using Zoom software, and schedule participants for a focus group.

### Procedures

We conducted virtual focus groups using Zoom software in March and April 2023. Each focus group included one moderator and one notetaker. Two members of the research team trained in qualitative research methods alternated serving as the moderator (LMR, SAC), and three members of the research team alternated serving as the notetaker (SAC, SDK, CM). We conducted a total of 6 focus groups, each containing 5–7 participants, that lasted between 58 and 78 min. We recorded the audio and video of each focus group. The incentive for participation was a $50 Amazon gift card. This study adhered to the principles of the Declaration of Helsinki. Ethics approval for the study was provided by the University of North Carolina at Chapel Hill Institutional Review Board (Protocol #22-1858). Informed consent was obtained from each study participant before the focus group discussions.

### Stimuli

**Fig. 1:**
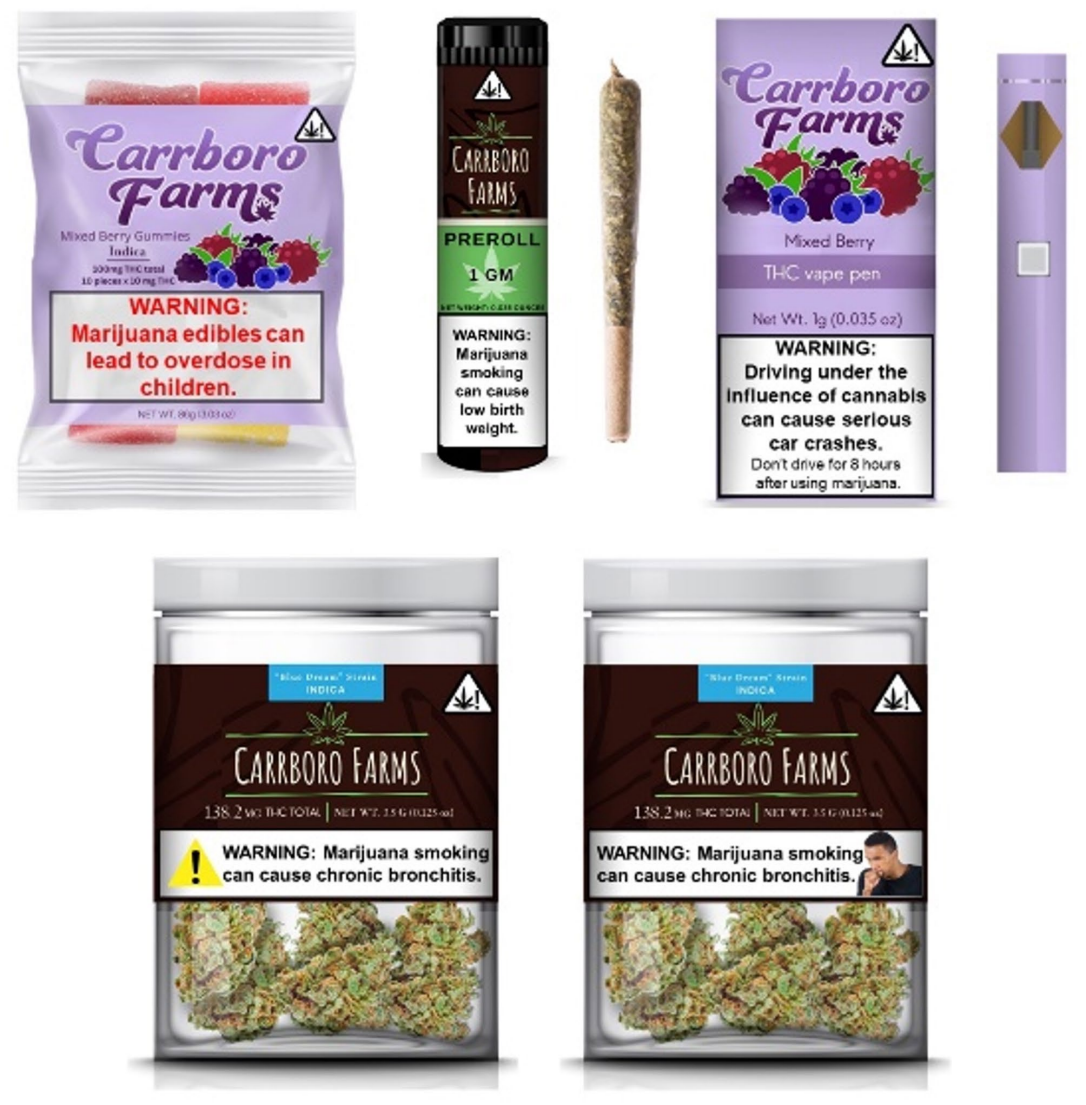
Novel cannabis warnings and packages created by the study team

We conducted a search of cannabis products from online retailers and contacted dispensaries and colleagues to locate images of cannabis packages and warnings. We received photos of cannabis packages with warnings from Alaska, California, Colorado, Illinois, Missouri, New Mexico, and Oregon. After viewing the photos, we selected three cannabis products (concentrate cartridges, loose dried flower, and edible gummies) with warnings reflective of existing warning content in the US (images not presented due to copyright law) [34]. We then developed four novel cannabis packages (edible gummies, pre-roll, THC vape pen, and dried loose flower) with warnings that varied *content* based on evidence about cannabis health effects [9] and *designs* based on best practices from tobacco, alcohol, and food warning label research [15, 19, 23, 35–37],. A research team member with experience in graphic design created the packages with a fictitious brand name (“Carrboro Farms”) to minimize the influence of brand loyalty and reputation on product perceptions [38]. Table 1 provides information about the focus group stimuli, and Fig. 1 presents the novel warnings we developed.

**Table 1:** Characteristics of Focus Group Stimuli

Type of Warning	Product	Warning Placement on Package	Icon/ Image	Text Color	Warning Statement
Existing	Vape pen cartridge	Back	No	Black	Marijuana has intoxicating effects and may be habit forming and addictive. Marijuana can impair concentration, coordination, and judgement. Do not operate a vehicle or machinery under its influence. There may be health risks associated with consumption of marijuana. For use only by adults twenty-one and older. Keep out of reach of children. Marijuana should not be used by women who are pregnant or breastfeeding. ​
Existing	Dried Cannabis flower	Side	Yes; Marijuana flower with adjacent exclamation point in a rectangle outline	White	**WARNING: ​**For use only by adults, 21 and older. Keep out of reach of children. Do not drive a motor vehicle while under the influence of marijuana. ​
Existing	Cannabis-infused gummies	Back	No	Black (with red marker word “WARNING” and “BE CAUTIOUS”)	**WARNING**: For use only by adults 21 and older. Keep out of reach of children. Do not drive a motor vehicle while under the influence of marijuana. This Product is not approved by the FDA to treat, cure, or prevent any disease.​ **BE CAUTIOUS**: Cannabinoid edibles can take up to 2 hours or more to take effect. ​
Novel	Cannabis-infused gummies	Front	No	Red	**WARNING: Marijuana edibles can lead to overdose in children**
Novel	Pre-roll blunt	Front	No	Black	**WARNING: Marijuana smoking can cause low birth weight.**
Novel	THC vape pen	Front	No	Black	**WARNING: Driving under the influence of cannabis can cause serious car crashes.** Don’t Drive for 8 hours after using marijuana.
Novel	Dried Cannabis flower (V1)	Front	Yes; yellow triangle with black exclamation point inside	Black	**WARNING: Marijuana smoking can cause chronic bronchitis.**
Novel	Dried Cannabis flower (V2)	Front	Yes; image of young adult male coughing	Black	**WARNING: Marijuana smoking can cause chronic bronchitis.**

### Focus group questions

The research team, which included members with expertise in cannabis warning label research, developed the focus group discussion guide (see Appendix A) using an iterative process. The first set of questions addressed reasons for using cannabis, cannabis risk perceptions (in general and relative to tobacco and alcohol), awareness and recall of cannabis warnings, and suggestions for what should be included in cannabis warnings. The moderators then presented images of three existing cannabis warnings and asked about cognitive and affective reactions to the warnings. To stimulate conversation about other warning formats, we also presented each novel warning and again asked about cognitive and affective reactions. The final set of questions asked participants about the purpose of cannabis warnings and any potential unintended consequences.

### Analysis

The focus groups were professionally transcribed verbatim and imported into ATLAS.ti (version 23) [39]. We created a codebook by developing deductive codes from the focus guide topics that pertained to the research question, “How do people who use cannabis perceive existing warnings, describe product risks, and react to novel cannabis warnings?” To start, four researchers (LMR, SAC, KLJ, CJM) gained familiarity with the codebook and established protocols for coding, such as capturing ID numbers within the text excerpt. Next, coders refined the codebook in a three-step process and established inter-rater reliability. First, coders independently read and coded the same transcript and identified inductive codes for the codebook. Second, all coders met to review the coded transcript, resolve coding differences, and adjust definitions in the codebook for clarification. Third, after establishing interrater reliability, coders dual-coded the remaining five transcripts and resolved discrepancies in meetings. Inter-rater reliability across all dual-coded transcripts ranged from 0.73 to 0.93, indicating moderate to high reliability [40]. After all coding was completed, we performed “code and retrieve” analyses, sorting the resulting text excerpts by coded categories [41]. The research team discussed the findings and identified major themes from ideas and concepts that emerged using a thematic data analysis approach. Specifically, we searched for themes or patterns across the focus group discussions rather than within one focus group or from one person [42]. Our themes captured important information about the data in relation to the research questions. We relied on inductive coding techniques as described by Strauss and Corbin [43] along with the constant comparison method [44]. 

## Results

### Participant characteristics

A total of 36 people participated in our focus groups. Half of the participants (50%) identified as White, with the remainder identifying as Black or African American (22%), Asian (19%), more than one race (6%), or preferred not to report their race (3%). A quarter identified as Hispanic or Latino/a (25%). On average, participants reported using cannabis 3.7 of the past 7 days (SD: 2.3) and 13.1 of the past 30 days (SD: 9.8). In addition, based on discussions in the focus groups, a quarter of participants (25%) described using cannabis for medical reasons (e.g., anxiety, pain, sleep), while a third (33%) described using cannabis for both medical and recreational (e.g. for fun) reasons. Finally, participants resided across 14 different states, with the Northeast (44%) and West (42%) being the most represented (Table 2).

**Table 2:** Participant characteristics, *n* = 36

Characteristic	*N* (%)
Age (years), mean (SD)	30.5 (7.8)
Age (years), range: 21–52	
21–25	15 (42%)
26–34	9 (25%)
35–54	12 (33%)
55+	0 (0%)
Race	
Black or African American	8 (22%)
Asian	7 (19%)
More than one race	2 (6%)
White	18 (50%)
Preferred not to report	1 (3%)
Ethnicity	
Hispanic or Latino/a	9 (25%)
Gender	
Woman	21 (58%)
Man	12 (33%)
Non-binary or gender queer	3 (8%)
Sexual Orientation	
Gay or lesbian	6 (17%)
Bisexual	7 (19%)
Straight	20 (56%)
Used a different term (queer, pansexual)	3 (8%)
# of days used cannabis in past 7 days, mean (SD)	3.7 (2.3)
# of days used cannabis in past 30 days, mean (SD)	13.1 (9.8)
Frequent cannabis use (20 or more days a month)	12 (33%)
Reason for cannabis use	
^+^Recreational	14 (39%)
^±^Medical	9 (25%)
^∗^Both (recreational and medical)	12 (33%)
Unknown/Did not specify	1 (3%)
State, 14 states represented	
Northeast	16 (44%)
Midwest	5 (14%)
West	15 (42%)
South	0 (0%)

^+^Coded recreational if participant mentioned using for pleasure or enjoyment (fun, socially)

^±^Coded for medical if participant mentioned using for medical condition (anxiety, pain, or for sleep)

^∗^Coded for both if mentioned both medical and recreational reasons for use

### Overview of themes

Three central themes emerged from the qualitative focus group data about awareness, perceptions of, and reactions to existing and novel cannabis warnings and product risks. We present the central themes and provide illustrative and supportive quotes for context.

#### Theme 1: participants expressed that the purpose of warnings should be to educate or promote safe use

Most participants expressed that the purpose of cannabis warnings should be to inform and educate potential consumers on how to use cannabis safely and responsibly, *“I would say to inform people of the health risk so they can make an educated decision as to whether they want to do it themselves”* (man, age 25). One participant expressed, “*Ithink a warning can be an educational moment on marijuana containers”* (woman, age 25), while another commented that the purpose of cannabis warnings should be *“To use responsibly. It doesn’t have to be to completely dissuade people from using it”* (woman, age 34). Several participants pointed out the need for inexperienced cannabis users to be informed about what to expect when using cannabis and how to use it safely, *“I love the idea that it’s instructional* [referring to the novel gummies warning] *to someone who doesn’t know. And I think that’s for me […] the biggest concern is to the people who are new users who don’t know*,* right? And then they like overdose cause they decide to eat like four gummies or something […]*” (woman, age 36). Participants expressed that the information shared should also be credible, *“[warnings] should be informing the person but again*,* in order to inform them*,* it has to be like vetted information […] should be based off of like scientific facts that we can all agree on”* (woman, age 24). Some participants felt that warnings should discourage certain populations (e.g., minors, pregnant people) from using cannabis, *“The goal is to ensure that marijuana doesn’t end up in the hands of those it shouldn’t*, i.e.,* children*,* teenagers*,* those who are under 21*,* and also warnings for safe*,* beneficial use for all those who qualify to use the product”* (woman, age 52).

A few participants had strong opinions that warnings are a strategy to limit industry responsibility for any negative events resulting from cannabis use, “*Well*,* I think it also has to do with making the companies not liable for people’s health risks that come along in the future so that they can’t get sued”* (woman, age 33). Another participant agreed, “*I feel like the goal of warning labels in general in America is lawsuit prevention”* (woman, age 33).

When reflecting on the purpose of cannabis warnings, several participants stressed the importance of warnings accurately reflecting the health risks of cannabis use, rather than overstating the health risks. Some participants acknowledged the historical overcriminalization of cannabis, particularly for communities of color, and expressed concern that over-dramatized warnings may further perpetuate this perspective, “*I feel like with the push for drug decriminalization*,* [warnings] could fuel the argument that*,* see*,* it should be criminalized again because it does have these harmful effects*” (woman, age 23). Another continued that, “*I think after decades long racially-based campaigning to hurt large communities*,* I think you need to be really careful about warnings that use excitatory language […] have some intention with it to not cause additional harm to people and really focus the intention on the warning labels on people keeping safe during their process*” (man, age 30).

#### Theme 2: participants did not notice existing warnings and wanted warnings to capture attention

Before viewing any cannabis warnings, participants were asked whether they remembered warnings from cannabis products they used. Most participants reported that they could not recall specific cannabis warning labels, “*I’m sure there must be warnings because it makes sense that there would be*,* but I have never registered them […] I think I’m just not observant to them”* (non-binary, age 39) and *“I think on the gummies […] They have like some sort of something. I don’t ever pay attention to them. I just eat them”* (woman, age 21). Only a few participants recalled current warning statements, *“I do remember seeing warnings that say*,* do not use while pregnant for marijuana […]”* (woman, age 24) and *“For the gummies that look like Sour Patch Kids and stuff it says*,* keep away from children”* (man, age 21).

Focus group participants then viewed and commented on existing cannabis warnings. Almost all participants noted that existing warning labels on packages had small font sizes, lengthy text, and were not attention-grabbing, *“The font is really small and then just by looking at how many words and stuff is in it*,* like people are going to look past that. It looks almost like a paragraph. Am I gonna read that?”* (man, age 35), and *“Just small - I’d basically not even read it to be honest with you. […] it doesn’t grab my attention to read it as a warning”* (woman, age 52). One positive characteristic of existing cannabis warnings was noted by a participant and affirmed by others, *[…] But I like the fact that they put the word “Warning” and “be cautious” in red. So*,* I might skip reading the other things* [referring to existing cannabis infused gummy warning]*”* (woman, age 36).

Next, participants were shown novel warnings developed by the research team. Most participants responded positively about the large font, the use of color text, and the warnings presented on the front of the packages, *“I would say as far as the font size it’s good. Color*,* red is better for catching the eye*,* but the black contrast on a white background also works. And being upfront*,* definitely good”* (man, age 25). Placement in the front was also important to some participants, *“I think it being in the front in a different color than the labeling also would make me read it right away”* (woman, age 23) and *“I think it might be more important to put that label right on the front to really catch someone’s attention”* (woman, age 35). Additionally, most participants liked novel warning statements that were clear and concise and offered practical information, *“I definitely agree that keeping it concise is probably for the best”* (woman, age 34) and *“I like the more literal wording in the new warnings like we were talking about car crash and chronic bronchitis or physical conditions or incidents that could happen instead of more general terms.”* (woman, age 21).

Some participants liked including specific product guidance on warnings. For instance, in response to the novel vape pen warning, one participant remarked, *“I like how it gives you the warning*,* but it also gives you a guideline and regards to how much you should wait until it says don’t drive for eight hours after using”* (woman, age 25). However, some participants reacted negatively to some novel warning statements, particularly the novel gummy warning, *“[…] I think overdose is kind of a weird word to use here. […] When we think of overdose*,* my immediate thought is someone in respiratory arrest […] and as far as I know*,* with kids*,* they can just get*,* you know*,* really sleepy*” (man, age 36). Some participants thought this warning statement was not relevant to cannabis users, “*[…] but like the actual content could be better. Like this isn’t something that people think about*,* like children shouldn’t have [*cannabis*] regardless”* (man, age 38). Another participant shared, “*But […] you know college kids are gonna see that and be like*,* well there are no kids around here. […] So*,* to them*,* the warning is it’s dangerous to kids*,* it’s not dangerous to adults […]*” (woman, age 21).

Focus group participants provided suggestions to increase attention to future cannabis warnings, *“I would want some kind of a warning icon or symbol or something to be put next to [warnings] so it would actually prompt me to read the text […] like exclamation point with the marijuana leaf symbol.”* (woman, age 24). Another participant suggested, *“I think maybe they can start just using images […]”* (woman, age 30). Participants also had recommendations about potential content of the future warnings, *“It might be good to say like how long it lasts or how long it takes to come on if you’re gonna do like first-time use information”* (man, age 23). Another reflected on warning content themes, *“I kind of wish it said more about like psychological and physical effects*,* and how do you like dose it or […] what are like the harmful effects if you take too much”* (woman, age 26).

Many participants also recognized there is limited space on cannabis packaging for warnings and had unique suggestions to provide information, *“I think it’s best to have sort of a quick warning on the front that’s just sort of an overriding statement of*,* ‘This may cause health risks’ and then like more information on the back”* (woman, age 24). One participant proposed a way to format health risks in warnings, *“I would put bullet points*” and the use of a QR code, *“if someone wants more information about how it affects pregnancy*,* maybe they might scan that QR code and at least they can get more information”* (woman, age 36).

#### Theme 3: participants were aware of both the benefits and risks of using cannabis

Participants reported a variety of different reasons for using cannabis, including improved sleep, enriched social interactions, and increased energy, *“[…] I like the edibles that help me just like fall asleep”* (woman, age 50) and “*I’d say it’s a fun experience in the right settings*,* and I also do it socially”* (man, age 25). Other participants highlighted personal benefits, *“I found it to be a great weight loss supplement if you use it with exercise. You have more stamina; you have more strength […]”* (woman, age 52). Other participants reported medical benefits of cannabis, including pain management and reduced anxiety, “*[…] I like smoking it and getting high. But other than that*,* I smoke for my anxiety”* (man, age 35) and*“[…] it’ll help with the pain cause I’ll ingest it*,* but I’ll also use it topically and do tissue work. But it’ll also help calm me down when the pain gets me freaked out and anxious”* (man, age 30).

When asked to compare the relative risk of cannabis products to tobacco and alcohol, many viewed cannabis as the safer option, *“I definitely look at […] marijuana as like the safest out of the three.”* (woman, age 21). One participant commented that cannabis has health benefits unlike tobacco and alcohol, *“[…] like there’s a significant difference because marijuana has a lot of healing properties*,* and when you think about alcohol and tobacco*,* there’s nothing healing in that sense”* (woman, age 36). Further, participants perceived cannabis as having a lower potential for addiction than alcohol or tobacco, “*[…] I believe statistically marijuana is actually much lower than both tobacco and alcohol in terms of addiction potential*” (man, age 25).

While participants listed a variety of perceived benefits of cannabis use, some also acknowledged that cannabis is not without risks, *“You could also potentially green out*,* which is getting too high”* (woman, age 52) and *“I can get paranoid if I take too much*,* like too many edibles for example”* (man, age 25). Participants noted, in particular, potential mental health risks they had heard about or experienced, “*The same way it helps with anxiety*,* I feel like for some people it also causes anxiety.*” (woman, age 25) and *“…I’ll get some sort of like paranoia. It will like worsen anxiety symptoms sometimes.”* (woman, age 26) One participant felt warnings should highlight the risk of schizophrenia, *“I did mention like the schizophrenia risk… if you wanted to like warn someone*,* that would be a serious complication for sure.”* (male, age 25) Some participants acknowledged the risk of addiction with cannabis use, “*[…] be aware of chemical dependency because it is a drug so that’s one of the downsides”* (woman, age 24). A few participants expressed concern about the harms of high-potency cannabis, *“I feel like there should be tighter stipulations as to how it [high-potency cannabis] can be sold. […] it’s too potent so that people are not able to enjoy or be able to use it how it should be. It’s becoming more dangerous. […] I’m careful because the gummies that they’re making now are a bit too potent for it to be functional.”* (woman, age 52).

## Discussion

As more states legalize cannabis products for medical and recreational use across the US, examining how people who use cannabis describe product risks, perceive existing warnings, and react to novel cannabis warnings will aid in the development of future cannabis warnings. Our focus group conversations with people who use cannabis revealed a need for cannabis warnings to educate and protect consumers, increase warning attention through design modifications, provide more evidence-based information on risks, especially the risks of high-potency products, and be mindful of unintended consequences when creating warnings.

Uniformly, people who used cannabis perceived the purpose of cannabis warnings as a way to educate consumers on how to use cannabis responsibly and to dissuade select populations who should not use cannabis, such as underaged youth. People in the US are primarily exposed to positive messaging about the benefits of cannabis [6, 7], and information about cannabis harms is lacking [2–4]. Cannabis warnings offer an evidence-based way to increase public knowledge about cannabis harms [45]. However, it is important to note that (1) cannabis has known medical benefits and (2) the risks of cannabis are lower than other commonly used substances, like tobacco and alcohol [46], both of which were raised by participants in our focus groups. Consequently, the purpose of cannabis warnings may differ from other health warning labels. Research on the perceptions of cannabis warning labels suggests that people who use cannabis may be more influenced by messages focusing on safer use strategies, which is consistent with our focus group findings and an area for further research [47]. 

Our finding that existing cannabis warnings were not noticeable is consistent with prior research on cannabis warning recall [14, 45, 48], including a qualitative Canadian study reporting that people who use cannabis were generally indifferent to warning labels on cannabis packages [49]. Most focus group participants in our study reported that the existing cannabis warnings had small print and lengthy text. Future research to develop effective cannabis warnings may find that simply redesigning cannabis warnings with larger font sizes and reducing text can more effectively communicate product risks, similar to what we found in our study and what other warning label research has demonstrated [50–53]. Future research may also show that using contrasting colors and icons (e.g., symbols), which were perceived favorably by participants in our study, could improve attention, recall, and effectiveness of cannabis warnings [35, 52, 54]. Our study participants also liked concise, large warnings. Implementing short rotating warning statements, which is an evidence-based strategy for increasing attention to warnings, could allow for a wider breadth of cannabis warnings [55]. Finally, warnings could use marker words like “Warning” or “Be Cautious,” which were also perceived favorably by participants in our focus groups. While the research on marker words in tobacco and e-cigarette warnings is mixed [54, 56], one study found that e-cigarette warnings with marker words significantly improved recall for the warning when displayed in a different color [54]. Further research could explore the impact of marker words on cannabis warnings, including the impact of different marker words and how color may interact with the marker words.

Another way to improve warnings is by revising the content of warning messages. Participants emphasized that the credibility of cannabis warnings requires the content to be solidly based on up-to-date evidence of actual cannabis risks, avoiding fearmongering. For example, some participants perceived the word ‘overdose’ as over-dramatized in the context of the opioid epidemic and felt it undermined the credibility of the warning. Additionally, some participants felt that warnings about children or pregnancy were not relevant to them. Warning guidelines have stated that warning relevance and believability are important elements of effective warnings [55, 57]. Therefore, future cannabis warnings need to be tested with people who use cannabis to ensure they are believable and relevant.

Further, while some participants expressed awareness of the increased risk of psychosis associated with high-potency products, and concern about the lack of regulations for the sale of these products, most participants did not raise this concern. A recent review found that the use of higher-potency cannabis, relative to lower-potency cannabis, was associated with an increased risk of psychosis and cannabis use disorder [58]. Future cannabis warning research could explore effective ways to communicate about the risks of mental health effects when using cannabis products, especially high-potency cannabis. Finally, concerns were raised in our focus group discussion about government entities using warnings as a method to elude legal liability from potential negative outcomes of cannabis use. These concerns may stem from a growing public mistrust of the government over the past two decades [59]. A few participants noted that over-dramatized warnings without strong evidence could harm communities of color, which are disproportionately impacted by the historical overcriminalization of drug use [60, 61]. Future research should explore how the development of cannabis warning labels can avoid further perpetuating lingering harms resulting from the War on Drugs.

Our findings contribute to cannabis warning research in several ways. First, our study adds to the limited but growing field of cannabis warning research conducted in the United States [62]. Second, our findings elaborate on review studies suggesting that cannabis health warnings are an important strategy to educate people about the risks of cannabis [14]. This study also contributes to the growing research on pictorial cannabis warnings, suggesting that images may increase attention to warnings [48]. 

### Limitations

Our study has several limitations. While focus groups can offer important and insightful formative information, they have limited generalizability. Since our sample was restricted to adults who use cannabis, findings may not generalize to non-users of cannabis products. In addition, participants only viewed three existing cannabis warnings from two states and five novel warnings; therefore, results may not extend to all existing or other novel warnings. Also, interpretations of warnings may vary depending on whether people use cannabis for recreational vs. medical purposes. We did not recruit or stratify participants based on their reasons for using cannabis, nor did we explore whether reasons for use influenced perceptions of cannabis warnings. This could limit the generalizability of our findings and represents an area for future research. Finally, this study was also conducted remotely, so participants were unable to physically interact with the stimuli, which might have affected perceptions. In terms of demographics and cannabis use, our study was enhanced by recruiting a diverse group of people who use cannabis. Specifically, the composition of race and ethnicity in our focus groups was comparable to past-year cannabis use among people aged 12 and older [63]. Participants’ ages ranged from 21 to 52 with a mean of 30.5 years (SD: 7.8). According to the 2023 National Survey on Drug Use, young adults (age 18–25) had the highest (36.5%) past-year cannabis use [63], and the Monitoring the Future survey found that 42% of adults ages 19 to 30 reported cannabis use in the past year [64]. Over half of the participants in our focus groups were between age 21–34, which is representative of people in the US who have used cannabis in the past year.

## Conclusions

Our focus group findings suggest that cannabis warnings can be improved by using large, concise text warnings on the front of packages, incorporating icons and colors to increase attention, and providing evidence-based information on health-related risks. Cannabis warnings that inform, educate, and provide guidance for less risky use rather than deter product use may be well received by people who use cannabis. More research is needed on the evidenced-based health effects of cannabis use to aid in the development of cannabis warnings, particularly around mental health concerns of cannabis use.

## Electronic Supplementary Material

Below is the link to the electronic supplementary material.

Supplementary Material 1

Supplementary Material 2

Supplementary Material 3

Supplementary Material 4

## Data Availability

Availability of data and materials: The transcripts from this qualitative study are available from the corresponding author upon reasonable request.
