# Supplementary material for: Perceptions of cannabis warnings and recommendations for improvement: a qualitative study with people who use cannabis from the United States
Source: BMC Public Health. 2025 Jul 3;25:2363. doi: 10.1186/s12889-025-23518-1 (PMC12225255; doi:10.1186/s12889-025-23518-1)
Supplement: Supplementary file 1 — Supplementary Material 1 [file 12889_2025_23518_MOESM1_ESM.docx]

**Appendix A**

**Focus Group Guide**

**Greeting:** Welcome and Thank you for being here today. We really appreciate you taking the time to participate in this group discussion.

**Role:** My name is . I will be the moderator for today’s discussion. My role will be to ask some questions and to keep the conversation going. Today’s discussion will be about warnings labels for cannabis, also known as marijuana, which includes products such as combustible weed and edible products like gummies and candies. This discussion will last about an hour. Feel free to ask questions if you have any along the way.

Our conversation today is being recorded to help us remember what you said. Is that okay with everyone?

You may ask me to turn off the recorder at any time, or just say you do not want to answer a question. Everything said here today will be kept confidential and will be used only to help us in our research. Nothing you say will be connected with your name.

We have a lot to cover, so I may need to change the subject or move ahead with the discussion. But please stop me if you want to add anything or if you have any questions.

We are fortunate to have some help today. I’d like to introduce you to our co-moderator ______ and note-taker _______. They may ask some clarifying questions as they come up and take notes during the discussion.

**Your participation in today’s focus group will help us develop potential warning labels for marijuana products to help people understand the potential effects of using the product. Any questions before we begin?**

We will start our focus group today with a Round Robin where everyone provides an answer to the same question. This ice breaker will help everyone become more comfortable speaking on zoom.

**Section I: Pre-existing knowledge and risk perceptions of cannabis products**

1. What are some of the reasons you use marijuana products?
2. I would like each of you to tell me what you know about using marijuana? What are some benefits? What are some drawbacks?

**Probe:** Does using marijuana products help with health or non-health related problems? Which problems? Does using marijuana cause any health or non-health related problems?

1. When thinking about alcohol and tobacco, how would you compare the risks associated with these products and marijuana?
2. Tell me what marijuana products you prefer to use?

**Probe:** Are any marijuana products perceived as more harmful or less harmful?

**Section II: Perceptions of existing cannabis warnings**

Thank you for a great opening discussion, now I would like to move the discussion into marijuana warnings labels. Warnings are statements that you might see on tobacco packages or alcohol bottles about the risk or harms of the products.

1. Tell me what you think about warnings on marijuana package?

**Probe:** Do you think marijuana products need warnings? Why or why not would warnings be useful?

1. Can anyone tell me what warnings, if any, you remember seeing or reading on the marijuana packages they have used?

**Probe:** Where have you noticed warnings on your marijuana packages? Front/back of package? Length of text? Any image? What content or themes do you recall on marijuana warnings? In other words, what were the warnings about?

1. If you could create your own warning for marijuana packages, what warning would you like to see?

**Probe:** What would the warnings say? Where would the warning be located? Images?

**Section III: Show participants existing cannabis warnings.**

When I share my screen, you will see a current marijuana warning. Can everyone see the marijuana warning on the screen?

Please take a minute to read and view the warning on the screen. (**Repeat items for each of the 3 existing warnings**)

1. Please tell me what you think about this warning?

**Probe**: How does the warning make you feel? Annoyed, unconcerned, skeptical, irritated, nervous, hesitant angry, scared? Is this new information about marijuana? Who might you talk to about this warning? Would you talk to someone about this warning if they wanted to try marijuana, or to better understand the warning yourself?

**Section IV: Reactions to novel cannabis warnings**

Now let’s turn your attention to marijuana warnings that are currently not being used. When I share my screen, you will see more marijuana warnings. I did not develop these warnings, so please feel free to share your honest opinions about them. Can everyone see the marijuana warning on the screen?

Please take a minute to read and view the warning on the screen. (**Repeat items for each of the 4 novel warnings**)

1. Please tell me what you think about this warning?

**Probe**: How does the warning make you feel? Annoyed, unconcerned, skeptical, irritated, nervous, hesitant angry, scared? Is this new information about marijuana? Who might you talk to about this warning?

**Probe**: What did you think about the format of the warning? Color? Icon? Image? Primary and secondary message?

**Stop: Ask this question for the slide with two novel warnings only!**

1. When comparing these two warnings, what do you like or not like about each warning?

**Stop: Ask this question for the last slide with the existing and novel warnings**

1. Please describe what you like or dislike when comparing the current warnings to the new warnings? (**Ask only after all novel warnings are viewed**)

**Section IV: Unintended consequences of the novel warnings**

We have discussed some great ideas today. Lastly, I would like to finish by asking about the goal of marijuana warnings and any unintended consequences.

**Probe (if necessary):** In other words, it is a situation where an action results in an outcome that is not what was intended.

1. What do you think the goal of marijuana warnings is or should be?
2. What (if any) unintended consequences might result from any of the marijuana warnings we presented today?

I know your time is valuable, and we really appreciate hearing your ideas and opinions today. Before we end the focus group, is there anything else you would like to contribute to this discussion?

Thank you all so much for participating in this focus group today. Your incentive for participating today will be a $50 Amazon gift card sent to you by email.
