# Supplementary material for: Perceptions of cannabis warnings and recommendations for improvement: a qualitative study with people who use cannabis from the United States
Source: BMC Public Health. 2025 Jul 3;25:2363. doi: 10.1186/s12889-025-23518-1 (PMC12225255; doi:10.1186/s12889-025-23518-1)
Supplement: Supplementary file 2 — Supplementary Material 2 [file 12889_2025_23518_MOESM2_ESM.pdf]

Title: Perceptions of cannabis warnings and recommendations for improvement: A qualitative study with people who use cannabis from the United States

Version 1, Reviewer 1:

Date: 30 Sep 2024

Overall labelling of consumer products is an important topic especially when there is a potential for risk to health.

This small scale study took a rigorous qualitative approach to investigating this topic.

What is less clear is how this fits with an extensive literature on both consumer packaging and specifically cannabis products packaging and risk.

Introduction:

It might have been helpful to have contextualise this work in relation to other research on package warnings, for example alcohol and tobacco.

It also seems that the literature relating to risk could have been presented in more depth. It would also have been helpful to understand the use of warnings on cannabis based products in the context of the legal requirements for each state where participants were making their purchases.

I am not convinced that the aim to fill gaps in the literature has been achieved by proposed approach in this study.

Methods:

These are rigorous and the researchers have clearly attempted to objectively standardise the process by which stimuli were created. It would be interesting to have seen how closely these were related to actual warnings on products.

Results:

Whilst an n of 36 is reasonable for a thematic analysis I am not convinced that the data really reflect any conclusions in relation to filling gaps in the literature.

It would have been helpful to have had a more detailed profile of the cannabis users. Whilst the authors are careful to include recreational cannabis users many people reporting cannabis use are doing so both medically and recreationally.

A history of cannabis use prior to legalization would have given a better overview of the potential for individuals to make a risk calculation.
