# Supplementary material for: Perceptions of cannabis warnings and recommendations for improvement: a qualitative study with people who use cannabis from the United States
Source: BMC Public Health. 2025 Jul 3;25:2363. doi: 10.1186/s12889-025-23518-1 (PMC12225255; doi:10.1186/s12889-025-23518-1)
Supplement: Supplementary file 3 — Supplementary Material 3 [file 12889_2025_23518_MOESM3_ESM.pdf]

Title: Perceptions of cannabis warnings and recommendations for improvement: A qualitative study with people who use cannabis from the United States

Version 1, Reviewer 2:

Date: 09 Oct 2024

Comments:

1. Keywords: add health warning labels

2. The abstract (line 2) and introduction (line 45) mention the lack of qualitative perspective on cannabis warning labels. Are there any quant studies? Would be good to lay out what is known generally about cannabis warning labels and if there is a broader gap or simply with this kind of research.

3. Abstract: would add in the methods that the adults are all from states with legalized non-medical cannabis.

4. Line 81: the three existing warnings were chosen from Alaska and Oregon. Why was this the case? Need to explain not only why these were the jurisdictions but also how you chose those 3 warnings and why. Especially given that such a large percentage of participants were from the Northeast would have been important to include one from a state they might have been more familiar with. Would list this as a limitation and would include more about how these were chosen. It is unclear whether these were images that had average size text and average length of content, if they were longer or shorter... seems like it could lead to bias in the results.

5. Line 84: similarly, more information is needed about how the 5 novel warnings were developed. Why were those health themes chosen? Was this based on available scientific literature on health risks and harms?

6. Lines 111-112: more information on what thematic data analysis is or include citations/references.

7. Would be good to include additional reflections on participant characteristics and what this may imply in the results especially that related to age, gender and geographic location.

8. Line 136: Not sure that the theme adequately reflects what the quotes say. There are some discussing education and some safety but not discussing a balance. Suggest changing the wording of the theme, maybe its a spectrum between education and safety. This also needs to be reviewed in the discussion (Line 263).

9. For both sections "Theme 1" and "Theme 3", it is hard to follow the text. Would be helpful to break up the single paragraph and try to divide the themes further. Lack of clarity in both of these sections given the continous sentence + quotes structure.

10. Line 167: For Theme 2, need clearer divisions between the different themes touched upon in this section: remember warnings, shown real warnings and shown fake warnings. Hard to notice when you are transitioning from one to another and which quotes apply to which section.

11. Mention of a lack of cannabis naive individuals in the interviews would be important, perhaps as a limitation. Research into warning labels in other areas such as alcohol and tobacco looks at how these warning labels affect non-users, a key component of why warnings exist in the first place. For cannabis it is important that labels also work for those who should not be using (e.g. youth, children). Even if not included in the research, this is an important consideration and should be mentioned in the paper.
