# Supplementary material for: Perceptions of cannabis warnings and recommendations for improvement: a qualitative study with people who use cannabis from the United States
Source: BMC Public Health. 2025 Jul 3;25:2363. doi: 10.1186/s12889-025-23518-1 (PMC12225255; doi:10.1186/s12889-025-23518-1)
Supplement: Supplementary file 4 — Supplementary Material 4 [file 12889_2025_23518_MOESM4_ESM.pdf]

Title: Perceptions of cannabis warnings and recommendations for improvement: A qualitative study with people who use cannabis from the United States

Version 2, Reviewer 1

Date: 25 Mar 2025

Thank you for your revisions.

Overall the work is interesting and this approach to the growing field of legal cannabis has an important role o play in making cannbis use safer. However, this study is really limited in its scope.

The introduction still needs to be expanded. The authors in their response to reviewer's state that:

"Our study contributes to several research gaps. First, most cannabis research has been conducted in Canada; thus, more cannabis research is needed in the US.":

This statement is incorrect and shows a lack of understanding of the depth and breadth of cannabis research in the US, especially post legalisation.

The lack of background information on the participants still makes it hard to really apply the results beyond the focus groups in the study. Motives for use for example medical versus s recreational would make a an impriotnat difference in how packaging might be perceived.

My suggestion would be to use this work as a pilot for a more extensive and rigorous study.
